# The Hemodynamic Profile and Intraoperative Bleeding Impact on Liver Transplant Patients

**DOI:** 10.3390/jpm14030288

**Published:** 2024-03-08

**Authors:** Lavinia Nicoleta Brezeanu, Cosmina Elena Jercălău, Catalina Liliana Andrei, Octavian Ceban, Gabriela Droc

**Affiliations:** 1 Department of Anaesthesia and Intensive Care I, Fundeni Clinical Institute, 022322 Bucharest, Romania; gabidroc@gmail.com; 2 Department of Cardiology, “Bagdasar Arseni” Emergency Hospital, “Carol Davila” University of Medicine and Pharmacy, 050474 Bucharest, Romania; cosmina-elena.jercalau@drd.umfcd.ro (C.E.J.); catalina.andrei@umfcd.ro (C.L.A.); 3 Economic Cybernetics and Informatics Department, The Bucharest University of Economic Studies, 010374 Bucharest, Romania; octavianceban1995@gmail.com; 4 Department of Anaesthesia and Intensive Care, “Carol Davila” University of Medicine and Pharmacy, 419291 Bucharest, Romania

**Keywords:** liver transplantation, hemodynamic monitoring, PiCCO system, 30/90-day survival probabilities

## Abstract

Liver transplantation is the treatment of choice for end-stage liver disease and despite accumulated experience over the years, improved surgical techniques, better immunosuppression and adequate intensive care management, it still represents the greatest challenge for anesthesiologists. The aim of the study was the characterization of the hemodynamic profile of patients with liver cirrhosis undergoing liver transplantation with the help of the PiCCO system during the three surgical stages, the impact of bleeding on hemodynamic status and correlation between the amount of bleeding, lactate levels, severity scores and survival rate and complications. Another focus of this study was the amount of transfused blood products and their impact on postoperative complications. Our study included 70 patients who underwent liver transplantation in our center and were hemodynamically monitored with the PiCCO system. Data were processed using the Python 3.9 programming language. Results: The mean MELD severity score was 18 points. During surgery, significant variations in the hemodynamic parameters occurred. All patients had a decrease in cardiac output in the anhepatic phase, with 50% presenting a decrease of more than 40%. In total, 78% of patients showed a decrease in the global ejection fraction, with a median value of 30%.Overall, 75% of patients had a total blood loss of less than 6000 mL and 31 patients developed immediate postoperative complications with a 50% probability with blood loss exceeding 6500 mL. Seven patients (10%) did not survive after 30 days. An amount of 5 mmol/L of serum neohepatic lactate determines a 50% probability of complications. Conclusions: Surgical technique causes an important decrease in cardiac output. Intraoperative bleeding has a major impact on outcome and the first month represents a critical period after liver transplantation. Statistical tests describe the probability of 30/90-day survival and the occurrence of complications according to variables such as intraoperative bleeding and MELD severity score. Intraoperative transfusion correlates with the occurrence of postoperative complications.

## 1. Introduction

Liver transplantation is the treatment of choice for end-stage liver disease. Since the first successful liver transplant in 1967, the survival rate has improved considerably [1,2]. Despite numerous developments in surgical techniques, immunosuppression as well as perioperative anesthetic management, anesthesia for liver transplantation is considered the greatest challenge for anesthesiologists. It requires extensive experience as well as good knowledge of the pathophysiology of liver disease and its systemic manifestations. The cirrhotic patient is a high-risk patient with alterations in cardiovascular, pulmonary, hepatic and renal systems who also presents with coagulation disorders.

Cirrhotic patients usually present with a hyperdynamic status, with increased cardiac output and decreased systemic vascular resistance. Some patients present cirrhotic cardiomyopathy with impaired contractility and prolonged QT [3,4]. An early diagnosis of hemodynamic instability during liver transplantation as well as its prompt management are two of the most important factors for a favorable prognosis. Various modalities have emerged over time for adequate cardiac output monitoring [5]. The pulmonary artery catheter (Swan Ganz) was initially used, but studies have shown that it is no longer the gold standard for cardiac output monitoring in liver transplantation [6]. 

No standards exist for hemodynamic monitoring during liver transplantation, but there are a series of options, each with their advantages and disadvantages [7]. The choice depends on the center where the surgical intervention takes place, team experience, economic status, preoperative evaluation and optimization of the patient. The anesthetic consultant makes a personalized decision for appropriate hemodynamic monitoring during liver transplantation.

Our transplant center routinely uses the PiCCO system for hemodynamic monitoring during liver transplantation. It allows cardiac output determination that combines pulse contour analysis and the transpulmonary thermodilution technique. A femoral arterial thermodilution catheter needs to be inserted. One of the advantages is the continuous CO monitoring with pulse contour analysis [8]. Compared to a Swan Ganz pulmonary catheter, the PiCCO system is a less invasive method.

The hemodynamic status of the patient varies between the three important stages of the liver transplant surgery (preanhepatic, anhepatic and neohepatic) and the surgical technique. Preanhepatic stage is the initial stage of liver transplantation where liver dissection takes place. It is associated with a higher risk of bleeding when the patient presents with a history of surgical interventions, previous spontaneous peritonitis, redo liver transplant, portal hypertension or hypervolemia [9].

The anhepatic phase is characterized by the clamping of the portal vein, hepatic artery and suprahepatic veins. It ends with the reperfusion of the graft via the portal vein, in the majority of cases, or hepatic artery. Depending on the surgical technique used, the anhepatic phase can lead to cardiovascular instability due to the blockage of hepatic outflow or inferior vena cava clamping. Severe cardiovascular collapse can be managed with the use of vasoactive drugs, temporary portosystemic shunt or veno-venous bypass [10].

In the neohepatic phase, the graft is being perfused and the new liver slowly restarts its function. During this phase, the postreperfusion syndrome can occur and is characterized with a 30% decrease in arterial pressure for at least a minute during the first 5 min after graft reperfusion [11].

## 2. Materials and Methods

In this paper, we sought to elucidate some aspects related to the hemodynamic profile of patients with liver cirrhosis and its modification during the three important surgical stages. The objectives of the present study included the characterization of hemodynamic disturbances encountered during liver transplantation with the help of the PiCCO system and their variations during the three surgical stages, the impact of bleeding on hemodynamic status and correlation between the amount of bleeding, lactate levels and survival rate and complications. Another objective was the amount of transfused blood products and their impact on postoperative complications.

In order to achieve the research objectives, the present study included 70 consecutive patients who underwent liver transplantation in the liver transplant center of the Fundeni Clinical Institute between January and December 2022 and who were hemodynamically monitored invasively through the PiCCO (Pulse Contour Cardiac Output) system. The PiCCO system represents a standard monitoring system for adult liver transplantation in our hospital. Patients under 18 years of age or with incomplete data were excluded. The study was conducted in accordance with the declaration of Helsinki and approved by the Local Ethics Committee (no 16021/23.05.2023).

The inclusion of patients in this study was performed retrospectively and demographic data, laboratory data, severity scores, liver disease etiology, transfusion of blood products as well as hemodynamic parameters measured using the PiCCO device in the three surgical stages (CI, SVRI, GEF; GEDI) were recorded. Hospital protocol involves PiCCO calibration via the transpulmonary thermodilution technique with 20 mL cold saline during each of the three stages of liver transplantation and also every time new hemodynamic changes or instability occur. Recorded PiCCO parameters are averages of calibrated determinations in each of the three stages of liver transplantation. 

Postoperative data were also recorded, including the duration of postoperative mechanical ventilation, complications, intensive care unit length of stay and 30/90-day mortality.

For this group of patients studied, different variables that characterize the patients’ evolution during different surgical stages were analyzed. A series of variables were collected, including continuous variables (age, Meld severity score, creatinine, duration of surgery, bleeding, etc.), binary variables (sex; 30/90-day survival,) and categorical variables (diagnosis). In order to analyze the connections between these groups of variables, adequate statistical tests were performed. For binary variables, we used the statistical method entitled logistic regression through which we analyzed the way the explicative variable influences the probability of event occurrence expressed as the target variable. For example, the target variable may be the 30-day mortality and the explicative variable may be the Meld score. The logistic regression will estimate the impact of Meld score on the probability of 30-day mortality. Secondary to this estimation, a statistical model arises that explains the relationship between the Meld score and mortality. As a result of the transformations made, with the estimated coefficient and a Meld value, we can estimate the probability of mortality. The quality of this model depends on the Meld value implication in mortality, the number of patients and the sample quality. 

When the target variable is continuous, linear regression is used. In this case, the resulting coefficient secondary to logistic regression shows the marginal contribution of the explicative variable on the changes in the target variable. For example, for a coefficient of 5, for the explicative variable, if this increases by 1 unit, the target variable will increase by 5 units.

Every estimated coefficient is tested in order to see to which degree it will be significantly different from 0, meaning if the explicative variable has a real impact on the target value. For this case, we use the t test for linear regression and the z test for logistic regression. Data modeling was performed using the Phyton 3.9 programming language along with the classic modeling packages such as statmodels and the seaborn processing package.

## 3. Results

Our study included 70 patients, 38 men (54.2%) and 32 women (45.8%) diagnosed with liver cirrhosis and undergoing liver transplant surgery. The median age of the patients is 53.5 years. In total, 27% (19) of the transplanted patients were diagnosed with liver cirrhosis-HCV, 21.5% (15 patients) with liver cirrhosis HBV + HDV, 21.5% (15 patients) with toxic nutritional liver cirrhosis, 7 patients presented Wilson’s disease, and 2 patients underwent liver transplantation for acute liver failure.

**Figure 1:**
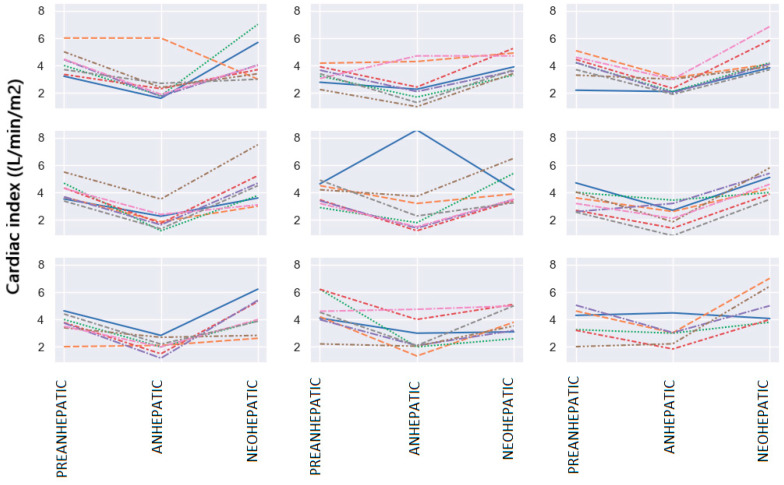
Cardiac index variation. Each line represents a different patient in the figure.

The mean Meld severity score for liver transplant patients was 18 points. The distribution of the Meld severity score is shown in Figure 1. In the present study, 10 patients were transplanted with a related donor liver segment, and the remaining 60 patients received a brain-dead donor whole liver. The median value of ascites fluid drained intraoperatively was 900 mL. In total, 50% of patients had less than 1000 mL of ascites fluid. The mean duration of surgery was 376 min. A proportion of 44.28% of patients (n = 31) developed complications in the postoperative period. 

Twelve (35%) patients developed pulmonary complications. Four patients developed pulmonary infection confirmed with signs/symptoms and chest imaging (CT scan or chest radiography) combined with inflammatory markers (C reactive protein, procalcitonin and presepsin), as well as positive cultures. Three patients had acute pulmonary noncardiogenic edema through the transfusion-related acute lung injury mechanism, which happened during blood product transfusion. Two patients developed pulmonary embolism confirmed with a CT scan. Three patients had significant pleural effusion on chest imaging, which needed chest drainage. Five patients developed surgical complications confirmed with a CT scan: Two patients had hemoperitoneum that needed surgical reintervention and three patients suffered complications with abdominal collections that needed CT-guided percutaneous drainage in the interventional radiology laboratory. Four (12%) patients had renal complications, leading to acute kidney injury stage III, which needed continuous veno-venous hemodiafiltration procedures. Three patients had cardiovascular complications: one presented with paroxistic atrial fibrillation on the electrocardiogram and two patients had non-ST elevation myocardial infarction confirmed with transthoracic echocardiography and dynamic changes in cardiac enzymes (hsTnI, CK and CK-MB). Seven patients developed hepatic complications and two patients had primary graft nonfunction (PNF) and liver function inconsistent with life that appeared in the first 7 days. According to strict UNOS criteria, PNF is defined as serum AST levels ≥ 3000 U/L associated with at least one of the following: INR ≥ 2.5, acidosis corresponding to arterial pH ≤ 7.30 or venous pH ≤ 7.25 and/or serum lactate levels ≥ 4 mmol/L [12]. Other clinical manifestations include persistent encephalopathy and metabolic acidosis, marked hypoglycemia, coagulopathy and reduced or absent bile production associated with a progressive increase in serum AST levels. Once the diagnosis was confirmed and other causes excluded, the patients were subjected to a redo liver transplantation. Two patients developed initial poor graft function, a less severe form of PNF. According to Olthoff et al. [13], using the Pittsburgh definition, initial poor graft function can be diagnosed by meeting at least one of the following criteria: bilirubin concentration ≥ 10 mg/dL on day 7, INR value ≥ 1.6 on day 7, and AST or ALT activity > 2000 U/L in the first 7 days after transplantation. Two patients suffered complications with hepatic artery thrombosis and one patient had right portal vein thrombosis. Three patients developed neurologic complications, including hepatic encephalopathy, which appeared in patients with graft dysfunction. Characteristics of patients who developed postoperative complications and of those who did not develop complications are presented in Table 1.

### 3.1. Variation in the Hemodynamic Parameters during the ThreeImportant Surgical Stages

The surgical technique used in our center consists of inferior vena cava clamping, including supra and infra hepatic clamping, in order to exclude the liver from circulation, which leads to significant venous stasis and compromising heart preload. During the surgical intervention, significant variations in the hemodynamic parameters were observed with a significant decrease in the cardiac index, the global ejection fraction and the global end diastolic volume in the anhepatic stage compared to the pre-anhepatic one.

- Cardiac Index (CI) Variation

The average value of CI in patients in this group in the pre-hepatic phase was 3.88 L/min/m^2^, 2.48 L/min/m^2^ in the anhepatic stage and 4.36 L/min/m^2^ in the neohepatic stage.

A decrease in the CI of approximately 38% is observed in the anhepatic stage as opposed to the pre-anhepaticstage. Also, in the neohepatic stage, there is an increase in the CI by approximately 11% compared to the pre-hepatic stage. The statistical analysis of the parameters measured using the PiCCO system showed that a value within normal limits of the CI (3–4 L/min/m^2^) in the pre-hepatic phase was present in 26 patients (37%), and an increased value of over 4 L/ min/m^2^ was present in 31 cases (44%). Figure 1 shows the variation in the CI along the three important surgical stages in the 70 patients undergoing liver transplantation.

**Figure 2:**
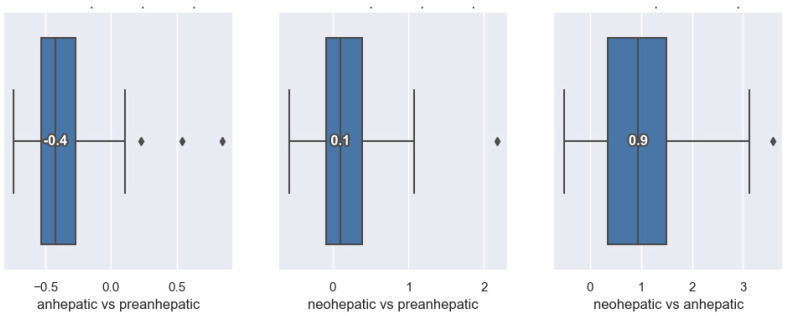
Distribution of cardiac index variation.

In the anhepatic phase, all patients showed a significant decrease in cardiac output. In this stage, compared to the pre-hepatic stage, 50% of patients had a decrease of up to 40% in the CI, and the remaining 50% showed decreases of more than 40%. The median value is −0.4, which represents a decrease of 40%. In the neohepatic stage compared to the pre-hepatic stage, 50% of patients had CI increases of more than 10%. In total, 25% of them showed a decrease in the CI, while the remaining 25% had increases of more than 30% (Figure 2). 

In the neohepatic phase compared to the anhepatic stage, it was observed that 50% of the studied patients showed an increase of up to 90% in the CI, and half showed an increase of more than 90% after declamping and reperfusion of the graft.

- Systemic Vascular Resistance Index (SVRI)

**Figure 3:**
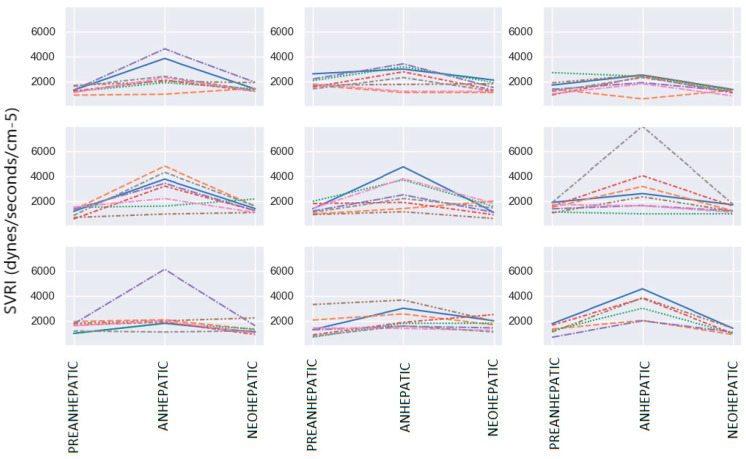
SVRI variation. Each color represents a different patient.

The mean value of SVRI was 1439 dynes/s/cm^5^/m^2^ in the pre-anhepatic stage, 2526 dynes/s/cm^5^/m^2^ in the anhepatic stage and 1083 dynes/s/cm^5^/m^2^ in the neohepatic stage. In the neohepatic stage, it was observed that SVRI does not return to the initial value but remains lower. Statistical analysis of the parameters showed that a low value of SVRI (below 1700 dynes/s/cm^5^/m^2^) in the pre-hepatic phase was present in 40 patients. Figure 3 shows the SVRI variation over the three important surgical stages in the 70 patients undergoing liver transplantation.

**Figure 4:**
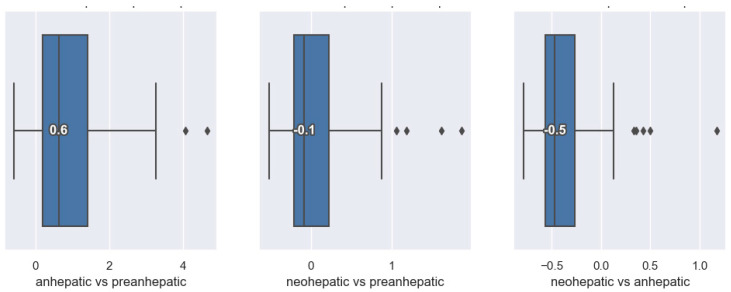
Distribution of SVRI variation.

In the anhepatic stage compared to the pre-anhepatic stage, more than 75% of patients show an increase in the SVRI. In total, 50% of patients show increases of more than 60% in the SVRI, while 30% of them show an increase of less than 60%. Less than 25% of the patients show a decrease in the SVRI in the anhepatic stage compared to the pre-anhepatic stage. In the last stage of liver transplantation, the neohepatic compared to the initial, pre-hepatic stage, no significant variation in the SVRI was observed, with 60% of patients registering decreases in the SVRI. In the neohepatic stage, 50% of patients show a decrease of up to 50% in the SVRI compared to the anhepatic stage, and the other half show a decrease of less than 50% (Figure 4).

- Global Ejection Fraction Variation

The average value of GEF in patients from the studied group was 33.7% in the pre-hepatic stage, 25% in the anhepatic stage and 34.5% in the neohepatic stage. The statistical analysis of the determined parameters showed that a normal GEF value (25–35%) in the pre-hepatic phase was present in 65 patients (92.8%), and a low value below 25% was present in 5 cases (7.2%). 

**Figure 5:**
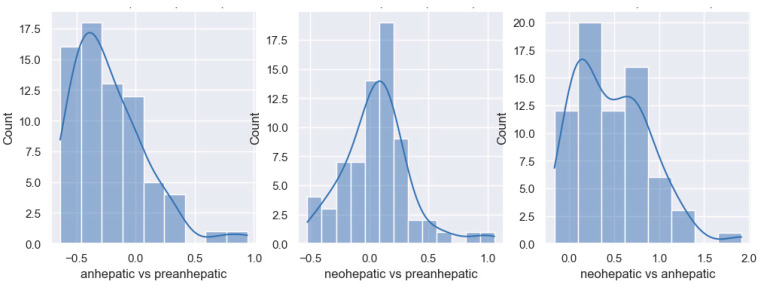
Distribution of GEF variation.

In the anhepatic phase compared to the pre-anhepatic one, half of the patients have a decrease in the GEF of more than 30%, and the other half have a decrease of less than 30%. In total, 78% of patients show decreases in global ejection fraction, with a median value of 30%. In the neohepatic stage compared to the initial stage of the transplant, the pre-hepatic stage, 60% of the patients show an increase in the global ejection fraction, with a median value of 10%. In the last stage of the transplant, the neohepatic stage, 85% of the patients show an increase of GEF compared to the anhepatic stage, with a median value of 40% (Figure 5).

- Noradrenaline Dose Variation

**Figure 6:**
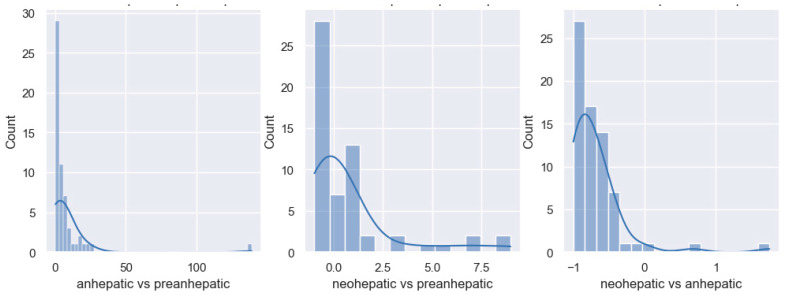
Distribution of noradrenaline variation.

The average value of the noradrenaline dose in the patients in the studied group was 0.22 mcg/kgc/min in the pre-anhepatic stage, 1.04 mcg/kgc/min in the anhepatic stage and 0.22 mcg/kgc/min in the neohepatic stage. Figure 6 shows the variation in noradrenaline dose along the three important surgical stages in the 70 patients undergoing liver transplantation.

In the anhepatic stage compared to the pre-anhepatic stage, all patients register increases in the need for vasopressor support with a median value of 380%. In the neohepatic stage compared to the pre-hepatic one, the dose of noradrenaline is similar to the initial one and the median value of the variation is 0, with approximately 25% of patients registering increases of 150–250% compared to the pre-hepatic stage. In the neohepatic versus the anhepatic stage, all patients experience decreases in noradrenaline dose with a median of 80% (Figure 6).

- Variation in Serum Lactate

**Figure 7:**
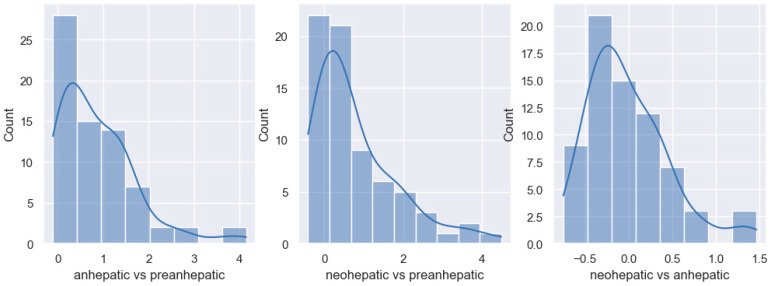
Distribution of lactate variation.

The average value of serum lactate in the pre-anhepatic stage is 2.49 mmol/L, in the anhepatic stage, it is 4.17 mmol/L, and in the neohepatic stage, the average value is 3.92 mmol/L. Figure 7 shows the variation in serum lactate along the three important surgical stages. 

In the anhepatic stage of the transplant, we observe that half of the patients show increases of over 70% in the serum lactate value compared to the initial stage. In total, 98% of all patients show increases in serum lactate at this stage. In the last stage of the surgical intervention, 75% of patients show increases in the value of serum lactate compared to the pre-hepatic stage, with a median value of 40%. The last phase of liver transplantation is characterized by a discrete decrease in the lactate value compared to the anhepatic stage (median value of −10%) (Figure 7).

### 3.2. Impact of Bleeding on Hemodynamic Status and Postoperative Course 

**Figure 8:**
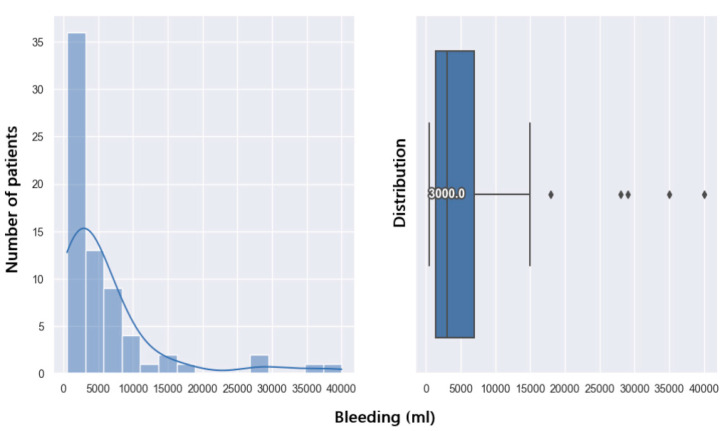
Bleeding distribution.

Median intraoperative bleeding is 3000 mL. In total, 75% of the patients in this study have a bleeding amount of less than 6000 mL. Following this graph (Figure 8), it appears that liver transplant patients have a 50% chance of losing an amount of blood below 3000 mL intraoperatively. 

**Figure 9:**
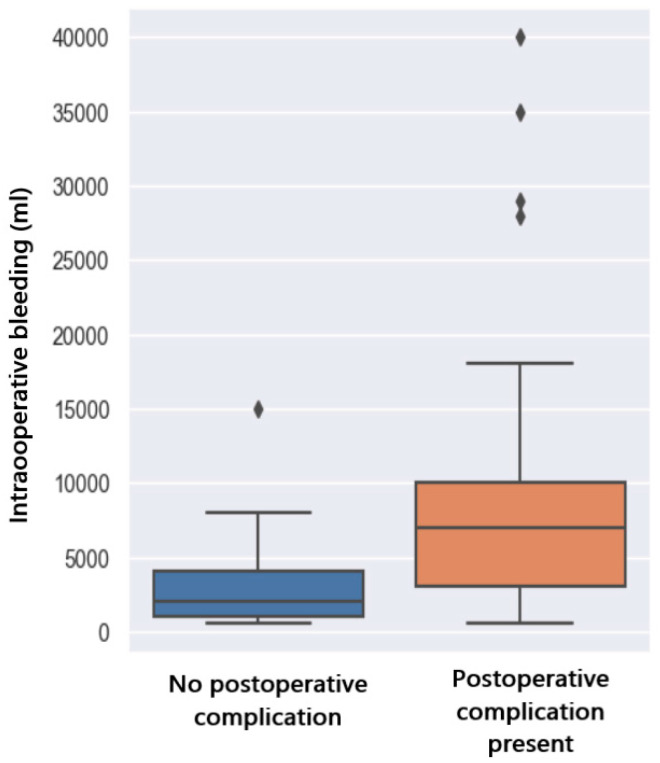
Complications related to bleeding.

From the group of patients studied, 31 of them (44.28%) developed immediate postoperative complications. From the distribution pattern below (Figure 9), it can be seen that patients who experienced postoperative complications had a higher degree of intraoperative bleeding. 

To observe the connection between these two variables, i.e., how the bleeding variable affects the postoperative complications variable, we use a logistic regression model. It can be seen that the complications variable is significantly different from 0 because the *p*-value is 0.002 (below 0.05). Thus, the probability of postoperative complications depending on the amount of bleeding is shown in the graph below.

**Figure 10:**
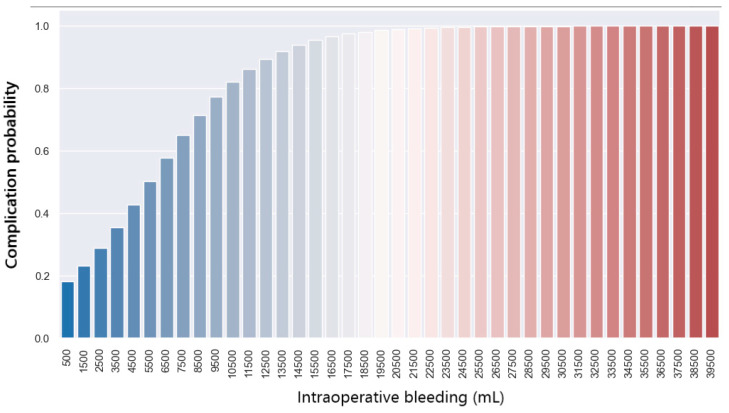
Prediction of complications with regards to bleeding.

From the graph below (Figure 10), it can be observed that there is a 50% probability of postoperative complications if the bleeding exceeds 6500mL. In case of a larger amount of bleeding, i.e., 22,000 mL, patients will have an 80% chance of complications after liver transplantation. 

**Figure 11:**
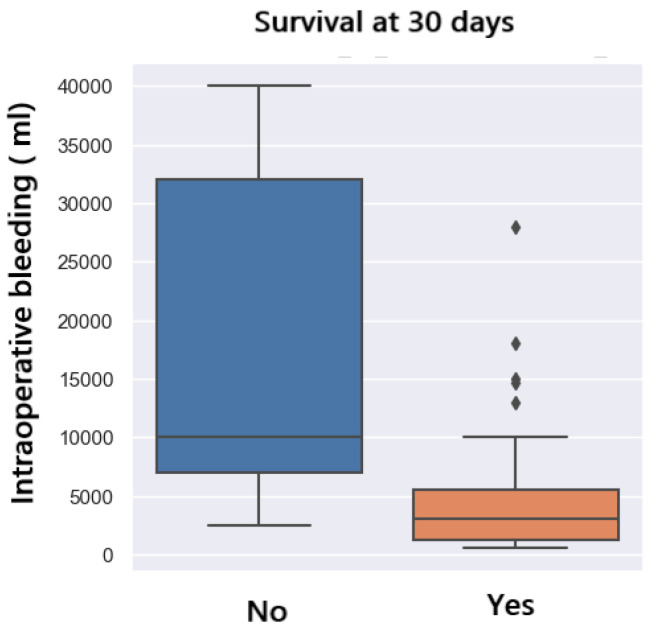
30-day survival related to bleeding.

In this study group, seven patients (10%) did not survive 30 days after liver transplant surgery. The analysis shows that patients who survive 30 days post-operation have a lower amount of bleeding (Figure 11).

**Figure 12:**
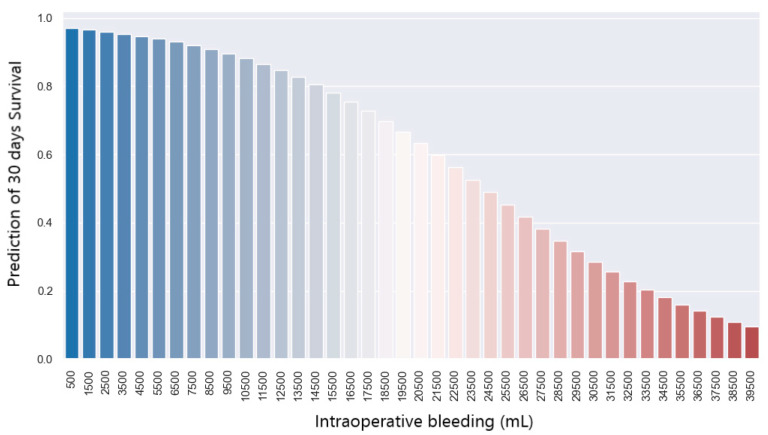
Prediction of 30-daysurvival with regards to bleeding.

To observe the connection between these two variables, i.e., how the bleeding variable affects the 30-day postoperative survival variable, we use a logistic regression model. We observe that the 30-day survival variable is significantly different from 0 because the *p*-value is 0.002 (under 0.05). Thus, the probability of survival at 30 days post-operation according to the amount of bleeding is shown in Figure 12. With a bleed of 10,000 mL, there is a 90% chance of survival at 30 days. In case of a larger amount of bleeding, i.e., 22,000 mL, the chances of survival at 30 days are reduced to 50%. 

In this study group, eight patients (11.42%) did not survive 90 days after liver transplant surgery. Most of the deaths, seven patients (10%), occurred within the first month; thus, this is the critical period after liver transplantation. 

**Figure 13:**
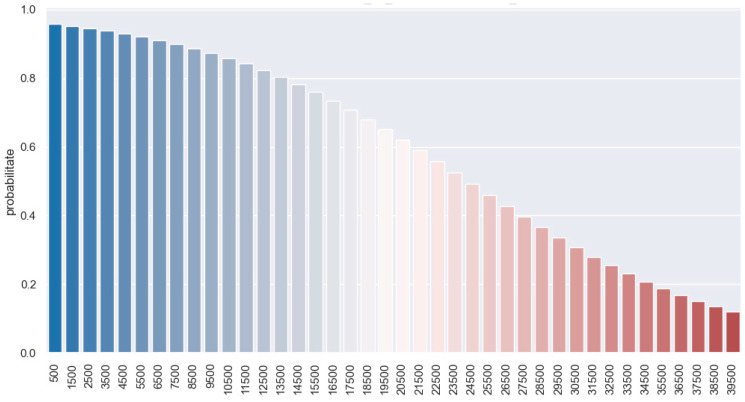
Prediction of 90-daysurvival with regards to bleeding.

To analyze the relationship between these two variables, i.e., how the bleeding variable affects the 90-day postoperative survival variable, we perform a logistic regression model. We observe that the variable survival at 90 days is significantly different from 0 because the *p*-value is 0.003. With a blood loss of about 5500 mL, there is a 92% chance of survival at 90 days. In case of a larger amount of bleeding, i.e., 10,000 mL, the chances of survival are reduced to 82%. A major bleed of about 20,000 mL will decrease the chances of 90-day survival by 50% (Figure 13).

Logistic regression was performed to confirm if intraoperative bleeding relates to the length of intensive care unit stay. It can be seen that the length of ICU stay is not influenced by the amount of intraoperative bleeding; it is not a statistically significant relationship (*p*-value 0.239).

Patients in the study group had a median Meld score of 19 points. A correlation between Meld score and intraoperative bleeding was observed. The logistic regression confirms the statistical importance (*p* = 0.01). Bleeding variation is explained by 9% of the preoperative severity score.

### 3.3. The Impact of Serum Lactate on Outcome of Transplanted Patients

**Figure 14:**
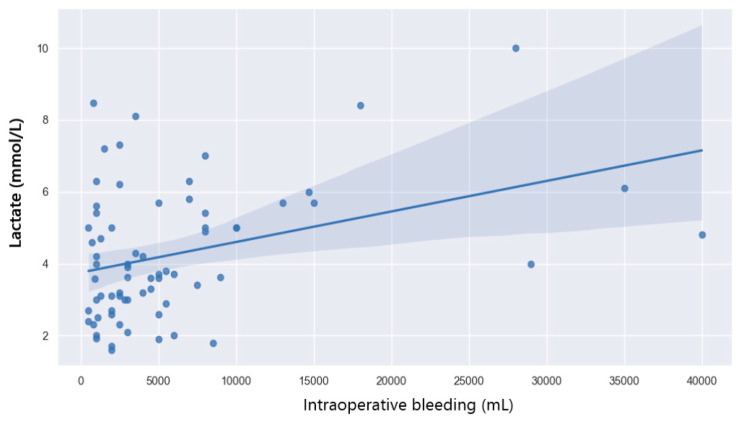
Correlation between anhepatic lactate and intraoperative bleeding.

The median value of anhepatic serum lactate is 3.8 mmol/L. In total, 50% of patients have serum lactate values above 5 mmol/L in the anhepatic phase. Figure 14 shows the correlation between anhepatic lactate and intraoperative bleeding. 

The logistic regression model shows that intraoperative bleeding is responsible for 13% of the variation in the anhepatic lactate level (*p* = 0.002).

The median length of intensive care unit (ICU) stay is 6 days. Anhepatic lactate correlates with the ICU length of stay. It increases by 0.53 each time the lactate increases by 1 unit. The logistic regression shows that the ICU length of stay is explained with a proportion of 9% by the anhepatic lactate variable (*p* = 0.012).

**Figure 15:**
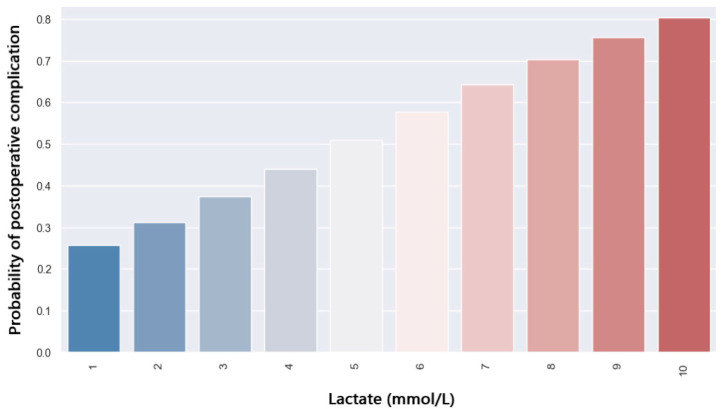
Probability of postoperative complication with regard to neohepatic lactate.

The present study also looked at neohepatic lactate and its impact on outcome. Neohepatic lactate does not correlate with the prolonged ICU length of stay (*p* = 0.17). Patients who developed postoperative complications had higher levels of neohepatic lactate compared to those without complications. The regression tests showed how neohepatic lactate affects the rate of postoperative complications (*p* = 0.033). The probability of complications depending on the value of neohepatic lactate is shown in Figure 15. A lactate level of 5 mmol/L is associated with a 50% chance of postoperative complications.

### 3.4. The Impact of Blood Transfusion on Outcome

**Figure 16:**
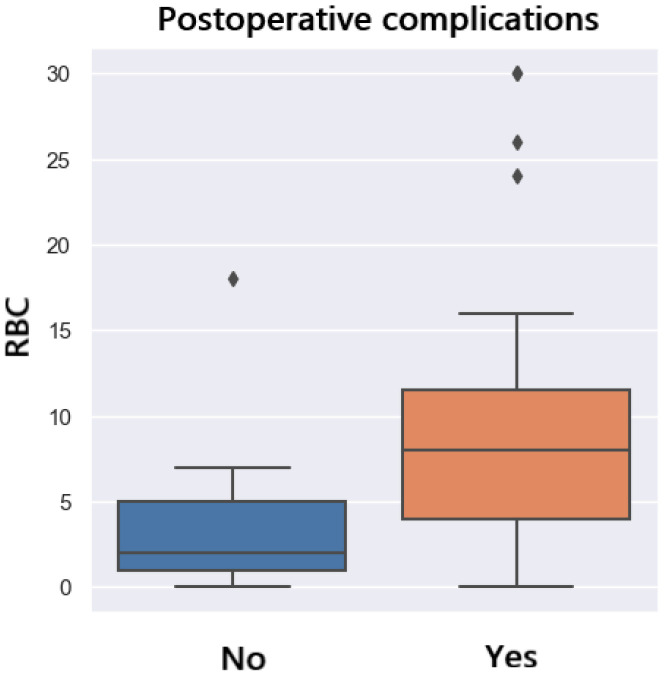
Complications related to RBC transfusion.

**Figure 17:**
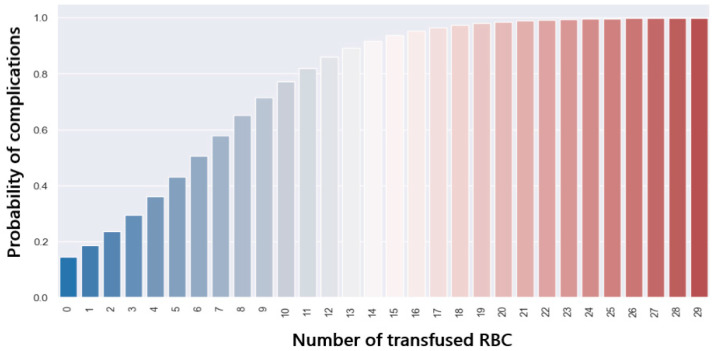
Probability of complications with regard to PRBC.

Figure 16 presents the distribution of complications in patients who received intraoperative packed red blood cell (RBC) transfusion. It can be observed that patients who developed postoperative complications had a higher amount of RBC transfused (*p* = 0.05). Intraoperative transfusion of 7 RBC leads to a 50% probability of development of postoperative complications (Figure 17).

**Figure 18:**
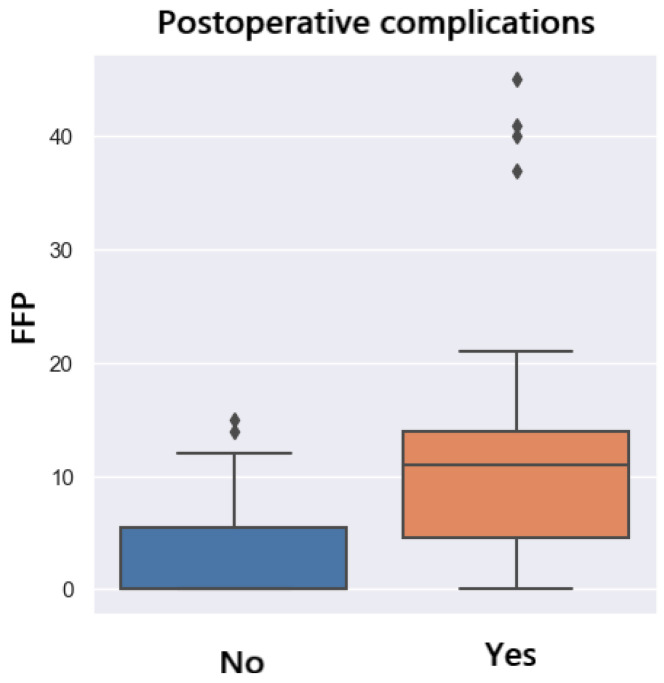
Postoperative complications and FFP transfusion.

**Figure 19:**
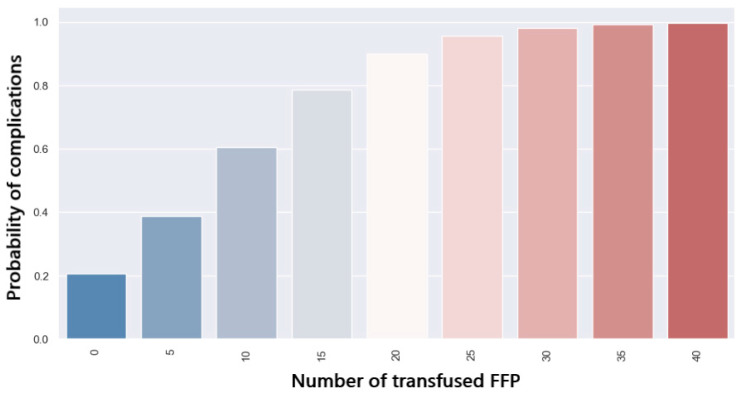
Probability of complications related to FFP transfusion.

Figure 18 shows the distribution of complications in patients who received intraoperative fresh frozen plasma (FFP). Patients who developed complications had a higher amount of FFP transfused (*p* = 0.001). The transfusion of 5 units of FFP will lead to a 40% chance of development of postoperative complications (Figure 19).

The same tests were performed for cryoprecipitate and platelets. Statistical analysis showed that there is no correlation between these blood products and the appearance of postoperative complications.

## 4. Discussion

Liver transplantation is the treatment of choice for end-stage liver disease. The Meld score is used all over the world for liver graft allocation. The present thesis reports the variation in hemodynamic parameters determined with the help of the PiCCO system during the three important surgical stages of liver transplantation. 

The literature presents various surgical techniques that can be used during liver transplantation [14]. The surgical technique routinely used in our center involves total IVC clamping with an important decrease in preload and cardiac output with implications for cardiovascular status and anesthetic management. The invasive cardiac output monitoring system is extremely important during liver transplantation due to the marked hemodynamic instability that can occur and can provide us with additional information related to cardiac output, dosing of vasopressor/inotropic substances and as guiding volume management and patients’ response to volume repletion. The study showed that in the anhepatic phase, there is an important decrease in the cardiac output, the global ejection fraction and the global end-diastolic volume. Half of the patients present a decrease in the CI with more than 40%. In the neohepatic stage, more than half of the studied patients present a value of cardiac output 10% higher compared to the value in the pre-hepatic phase due to the comeback of the venous return.

As a result of significant variations in CO, there are variations in SVRI. In total, 40 patients present a typical cirrhotic hiperdynamic status, with high CO and low SVRI values [15]. The anhepatic phase results in an important SVRI increase. The present literature advises using the piggyback technique for liver transplantation for better hemodynamic stability. It also suggests the use of veno-venous bypass in case of significant bleeding, hemodynamic instability or severe portal hypertension. One of the particularities of this study is the fact that our center does not use the piggyback technique; thus, hemodynamic instability must be accordingly managed by the consultant anesthetist.

Noradrenaline is the preferred vasopressor used and its variation is consistent with the hemodynamic status during the three surgical stages, including the anhepatic stage that requires high doses to maintain the mean arterial pressure and cardiac output [16].

Intraoperative blood loss can be secondary to impaired liver synthesis, portal hypertension, portal vein thrombosis or splenomegaly. All these factors associated with surgical technique can lead to significant blood loss and blood transfusion [17]. Despite recent progress, liver transplantation remains a surgical act with a high risk of bleeding [17]. 

Similar to the literature data, our study showed that intraoperative bleeding influences the outcome of transplant patients and there is a 50% chance of postoperative complications if bleeding exceeds 6500 mL. Most of the deaths occurred within 1 month postoperatively, which is the critical period after liver transplantation. With a blood loss of about 5500 mL, there is a 92% chance of 90-day survival. Intraoperative bleeding requires intraoperative transfusion with products such as PRBC, FFP, cryoprecipitate or platelets. Similar to the literature data, our study showed that RBC and FFP transfusion are associated with postoperative complications and prolonged mechanical ventilation. Due to the well-known side effects blood transfusion might have on patients, our center guides intraoperative transfusion with the use of viscoelastic tests—rotational thromboelastometry.

Statistical tests performed on this group of patients provided information related to the probability of 30/90-day survival and the occurrence of complications according to variables such as intraoperative bleeding and Meld severity score. Also, the anhepatic and neohepatic lactate values provide information related to the probability of complications and graft dysfunction. Increased intraoperative lactate values are a marker of tissue anoxia and liver graft dysfunction. Hyperlactatemia at the end of surgery has been demonstrated to correlate with an increase in 30- and 90-day mortality [18].

Studies show that the majority of complications occur during the first 3 months after liver transplantation [19].

The results of the present study open new hypotheses for future studies regarding hemodynamic monitoring of cardiac output during liver transplantation. The usefulness of transesophageal ultrasound in comparison with invasive means of monitoring of the hemodynamic status will be the focus of our next study.

## 5. Conclusions

The present paper investigates the hemodynamic status of liver transplant patients and their variation during the three stages of surgical intervention. The PiCCO system proved a useful tool for CO measurement and management during liver transplant. Considering the IVC clamping and the total blood loss, important hemodynamic variations were present during the three surgical phases, with the anhepatic stage being the most unstable with high noradrenaline doses. All patients presented a decrease in cardiac output and global ejection fraction in the anhepatic phase. Accurate hemodynamic management is necessary during this high-risk procedure on cirrhotic patients. Intraoperative bleeding has a major impact on patients’ outcomes and the first month represents a critical period after liver transplantation. Intraoperative transfusion correlates with the occurrence of postoperative complications. 

The present study has its limitations. It is a retrospective study and the total number of patients included is quite small. The lack of another cardiac monitoring device is another limitation of the study. 

## Figures and Tables

**Table 1:** Characteristics of patients with and without postoperative complications.

Variables	Postoperative Complications	
	YES	NO
Bleeding (mL)	9767 (10,099)	2900 (2726)
RBC (units)	9.5 (8.0)	3.1 (3.3)
FFP (units)	12.4 (12)	3.2 (4.4)
Platelets (units)	2. (2.8)	0.7 (1.9)
Cryoprecipitate (units)	1.9 (2.8)	0.6 (1.5)
Fibrinogen (g)	1.6 (2)	0.4 (0.8)
Cold ischemia time (min)	279 (142)	310 (102)
Warm ischemia time (min)	50 (28)	50 (20)
Total transplant time (min)	398 (90)	360 (85)
Reperfusion sindrom	23 patients	21 patients
Anhepatic lactate (mmol)	5.1 (1.9)	3.5 (1.3)
Preanhepatic lactate (mmol)	2.8 (1.3)	2.2 (0.9)
Neohepatic lactate (mmol)	4.7 (2.8)	3.4 (1.5)
CI preanhepatic (L/min/m^2^)	4.04 (0.96)	3.75 (0.86)
SVRI preanhepatic (dyne/s/cm^−5^)	1371 (485)	1488 (487)
GEF preanhepatic (%)	35.7 (8.9)	32.2 (5.4)
GEDI preanhepatic (mL/m^2^)	697 (181)	648 (152)
CI anhepatic (L/min/m^2^)	2.52 (1.09)	2.43 (1.31)
SVRI anhepatic (dyne/s/cm^−5^)	2165 (925)	2909 (1431)
GEF anhepatic (%)	25.6 (6.4)	24.2 (8.3)
GEDI anhepatic (mL/m^2^)	653 (178)	553 (131)
CI neohepatic (L/min/m^2^)	4.46 (1.2)	4.25 (1.08)
SVRI neohepatic (dyne/s/cm^−5^)	1335 (413)	1431 (336)
GEF neohepatic (%)	33 (6.1)	35.6 (6.6)
GEDI neohepatic (mL/m^2^)	720 (203)	673 (139)
Hemoglobin (g/dl)	8.2 (1.3)	8.1 (1.3)
Platelets (/mm^3^)	77,354 (59,452)	87,795 (98,606)
INR	2.13 (1.07)	1.9 (0.55)

Values represent mean (SD).

## Data Availability

The data presented in this study are available upon request from the corresponding author. The data are not publicly available due to ethical restrictions.
